# SPIN.DOC induces cellular transformation of NIH3T3 normal mouse fibroblast cells

**DOI:** 10.22099/mbrc.2025.52821.2125

**Published:** 2025

**Authors:** Khuraijam Mrinalini Devi, Thangjam Davis Singh, Rubismita Deka, Lisam Shanjukumar Singh, Thiyam Ramsing Singh

**Affiliations:** 1 Department of Biotechnology, Manipur University, Imphal -795003, India; 2 Department of Molecular Biology and Biotechnology, Tezpur University, Napaam, Tezpur 784028, Assam, India

**Keywords:** SPIN.DOC, NIH3T3, Oncogene, Cancer stem cell, Microtentacle

## Abstract

SPIN.DOC was discovered as a transcriptional co-repressor of wingless related integration site (WNT) pathway. However, it has been found to be upregulated in various types of cancer, including hepatocellular carcinoma, colorectal cancer, renal papillary cell carcinoma. Whether SPIN.DOC functions as an oncogene or tumour suppressor gene remains uncertain. Here, we report that ectopic expression of SPIN.DOC in normal NIH3T3 fibroblast cells promotes cell proliferation, colony formation, migration and invasion. Moreover, SPIN.DOC expressing NIH3T3 cells show increased spheroid formation, suggesting enhanced stemness and transformation potential. Immunofluorescence analysis using anti-β-Tubulin suggests that SPIN.DOC may induce, formation of tubulin-based microtentacles (McTNs), indicating epithelial-to-mesenchymal (EMT) transition. In conclusion, our study helps in establishing that SPIN.DOC can function as an oncogene.

## INTRODUCTION

SPIN.DOC is a Spindlin1 docking protein that modulates various transcriptional processes [1, 2]. Spindlin 1 functions as a histone methylation pattern reader with three tandem tudor like domain [3] and while SPIN.DOC, one of the Spindlin1- interacting proteins, act as a transcript-tional co-repressor by inhibiting its co-activator activity [4, 5]. 

Beyond this role, SPIN.DOC is involved in other cellular processes such as PARP 1-mediated PARylation in DNA repair [6], epigenetic regulation in germline development [7]. Initially identified as a transcriptional co-repressor of Wnt pathway, later studies suggest that SPIN.DOC is overexpressed in multiple tumour types. Despite its diverse functions, our primary interest is to determine whether it plays a role in tumorigenesis and can drive the transformation of normal cells into metastatic cells. For this transformation to occur, cells must detach from the surface and transition into circulating tumour cells (CTCs) [8]. During this process, the cytoskeleton network particularly microtubule, which are composed of α and β tubulin, plays a crucial role in metastatic invasion and cell migration [9]. 

Tubulin plays diverged roles in regulating of cancer progression, metastasis, aggressive behaviour, drug resistance or poor prognosis [10-12]. Additionally, several studies suggest that these tubulins are involved in the formation and maintenance of cancer stem cells (CSC) by regulating functionally essential components [13-18]. Given these findings, our focus is on understanding how the stable expression SPIN.DOC in normal cells influences various signalling pathways that drive cancer development and disrupt normal tubulin function. To investigate this, we conducted a series of assays- including cell proliferation, colony formation, cell migration, invasion and spheroid formation to assess the phenotypic and morphological changes in NIH3T3 cells upon SPIN.DOC expression. Collectively, our study provides a new insight into the cellular transformation of normal cells and the morphological changes associated with SPIN.DOC expression.

## MATERIALS AND METHODS

### Cell culture:

NIH3T3 mouse fibroblast cell was obtained from NCCS cell repository, Pune and cultured in DMEM medium (GIBCO), supplemented with 10% FBS (GIBCO, USA) and 1% penicillin- streptomycin (Thermo Fischer) at 37°C in a 5% CO_2_ incubator. All the experiments were performed using this NIH3T3 cell line. To ensure that the cells are free of mycoplasma, DNA staining with fluorescent dye were performed. 

### Transfection:

 1.5×10^6 ^cells were seeded in a 60 mm plate one day before the transfection. For overexpression of SPIN.DOC, transfection was performed using lipofectamine 2000 using the manufacturer’s protocol. At 60% confluency of the cells, 3 µg of the plasmid (pLVX-SPIN.DOC) was used for ectopic expression of SPIN.DOC while empty vector (pLVX) served as control. The cells were further seeded into vsrious wells or plates based on the requirement of the subsequent experiments.

### Cell proliferation assay:

24 hours post transfection, 15000 cells were seeded into a 12 well plate. Number of viable cells were counted at 24 h, 48 h and 72 h using the trypan blue exclusion method. The percentage of viable cells for each day was determined.

### Transwell migration and invasion assay:

Migration and invasion assay were performed using EMD Millipore QCM 24-well migration assay (ECM508) and CHEMICON cell invasion assay kit (ECM550), following the manufacture’s protocol. Briefly, 1.5 ×10^5^/ml of cells in serum-free medium were seeded in the upper chamber while 500 µl of complete medium containing 10% foetal bovine serum was added to the bottom chamber. Then it was incubated for 24 hours in a CO_2_ incubator. The non-invading cells were carefully scraped from the interior of the inserts with a cotton-tipped swab to avoid background staining. The insert was then stained, and the migrated and invaded cells were counted under a phase contrast microscope (Leica). Counting was done in triplicates to ensure statistically significant values.

### Immunofluorescence:

 Cells were cultured on poly-L-lysine coated coverslips and after 24 h, cells were fixed with 4% paraformaldehyde for 10 minutes. They were then permeabilized with 0.5% triton X-100 for 2 minutes, blocked with 5% BSA at 37°C for 45 minutes. Subsequently, the cells were incubated overnight at 4°C with the specific primary antibody. Then the cells were rinsed three times in PBS for five minutes each before being incubated with FITC-labelled secondary antibodies. Finally, coverslips were washed again, counterstained with DAPI and mounted. Then, they were analysed under a fluorescence microscope (LEICA).

### Colony formation assay:

NIH3T3/Control and NIH3T3/SPIN.DOC (200 cells each) were seeded in triplicate in a 6-well plate. Once the colonies became visible to unaided eye, they were fixed with 2% paraformaldehyde for 5 minutes and stained with Giemsa dye. Colonies containing 50 or more cells were counted either manually or using specialised cell counter software (Leica). 

### Formation of colonospheres/spheroid by Hanging Drop method:

 A restricted number of cells (200 cells per 15 ul drop) were cultured on the inner upper side of a 100 mm dish lid using a suitable spheroid-forming medium to generate primary colonospheres. It was performed in triplicate. After that, the lid was inverted and set above a plate containing 10 ml of PBS and incubated in hypoxia condition.

### 3D cell culture media with methyl cellulose:

Spheroid formation medium was prepared freshly before use by diluting methyl cellulose solution to 5 mg/ml in the appropriate culture medium (as described below) and passing through a 0.22 µm filter to sterilise and remove undissolved particles. Briefly, primary colonospheres were generated by incubating in serum-free stem cell culture medium (SCM) containing DMEM/F12 (1:1) supplemented with B27 (Invitrogen), 20 ng/ml EGF (Sigma, St Louis, MO), 10 ng/ml fibroblast growth factor (Sigma), insulin, 5 µg/ml and antibiotic-anti-mycotic in ultra-low attachment plates (Corning Inc, Lowell, MA) for 10 days. The colonospheres formed after 11 days were evaluated for their number and size by phase contrast microscope.

## RESULTS AND DISCUSSION

**Figure 1:**
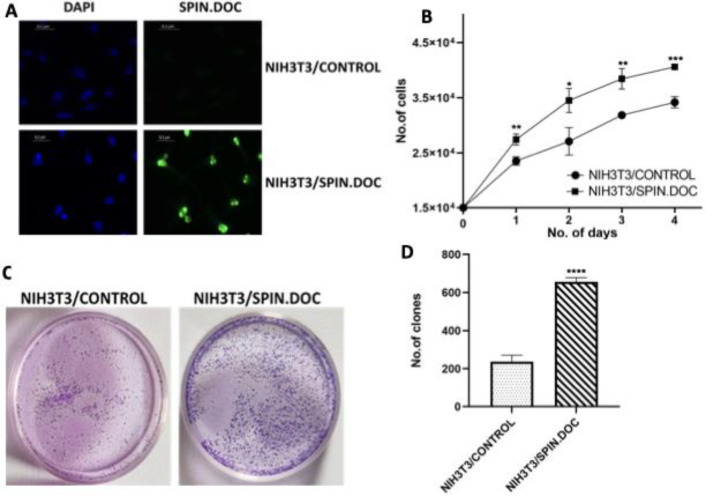
(A) Ectopic expression of SPIN.DOC in normal fibroblast NIH3T3 cells: NIH3T3 cells were either transfected with PLVX-SPIN.DOC or vector only. 2 days after transfection, expression of SPIN.DOC was analyzed by immunofluorescence using anti-SPIN.DOC and secondary antibody tagged with FITC. Scale bar, 0.2 µm. B) Cell proliferation of NIH3T3/Control and NIH3T3/SPIN.DOC groups were determined at the indicated days. (C) Representative images of Colony Formation Assay. Colony forming capacity of both the groups were determined following the materials and methods as given elsewhere. (D). Colonies with atleast 50 cells were counted. Data represented as the mean of ± SD of n=3 experiments; *, **, ***, and **** significantly different from normal control group indicates p< 0.05, 0.01, 0.001 <0.0001, respectively.

 SPIN.DOC was initially identified as an interacting partner of SPIN1 and forming a complex that acts as a transcriptional co-repressor of Wnt pathway. However, recent reports suggest that SPIN.DOC is overexpressed in various tumours, raising question on whether SPIN.DOC act as an oncogene or a tumour suppressor gene? To investigate this, we hypothesized that, if SPIN.DOC is an oncogene, its ectopic expression in normal cells should induce tumor like phenotypes. Given that NIH3T3 fibroblast cells are widely used due to their feeder layer ability, culture stability, and relevance in stem cell research, we chose them as a model system. We ectopically expressed SPIN.DOC in NIH3T3 cells. The expression of SPIN.DOC was confirmed by immunofluorescence using an anti-SPIN.DOC antibody. While the NIH3T3/Control cells showed no detectable immunofluorescence, the NIH3T3 cells transfected with SPIN.DOC (NIH3T3/SPIN.DOC) exhibited strong SPIN.DOC expression in all the cells (Fig. 1A). 

Proliferation assay based on cell counting at different time points, revealed that overexpression of SPIN.DOC in normal NIH3T3 cells, induced significantly higher cell proliferation, compared to the NIH3T3/Control cells (Fig. 1B). Additionally, colony forming assay demonstrated that SPIN.DOC expressing NIH3T3 cells exhibited significantly higher rate of clonal expansion than the control cells, further highlighting proliferative potential NIH3T3/SPIN.DOC cells over control (Fig. 1C and 1D). Together, these data suggest the role of SPIN.DOC in promoting cell proliferation and colony expansion, supporting its potential role as an oncogene.

**Figure 2:**
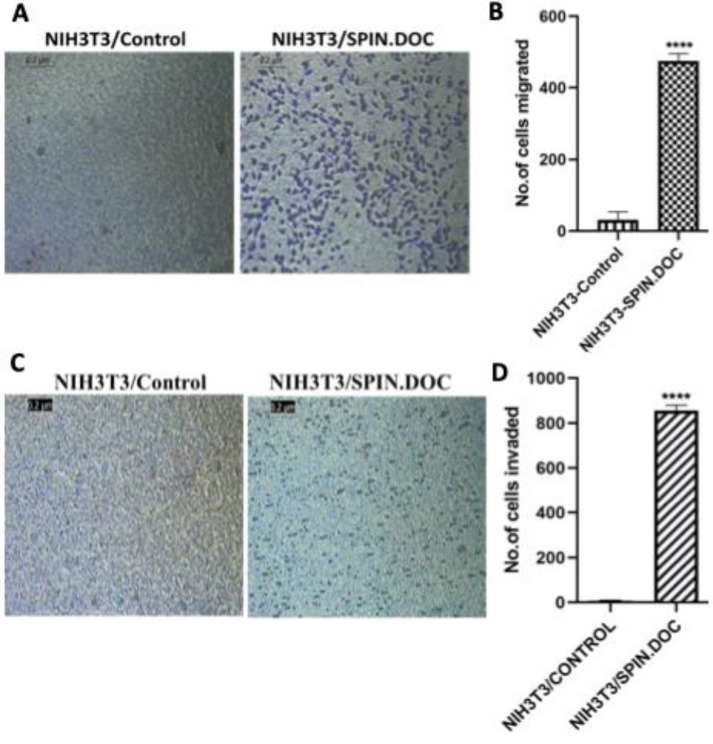
SPIN.DOC induced cells migration, invasion and microtentacle formation in normal NIH3T3 cells.

To further investigate the roles of SPIN.DOC in cell migration and invasion, we performed migration and invasion assays using the commercially available kits, following the manufacturer’s protocols, as mentioned in the materials and methods. Our results demonstrated that NIH3T3/control cells exhibited minimal ability to migrate through the Boyce chamber, whereas NIH3T3/SPIN.DOC cells showed a significantly higher migratory ability (*p*<0.0001). Interestingly, while NIH3T3 cells are typically non-invasive, a significant number of cells displayed invasive phenotypes upon SPIN.DOC expression (*p*<0.0001). Together, these data suggests an increase in the migratory and invasive competence of the NIH3T3/SPIN.DOC cell group as compared to NIH3T3/control group (Fig. 2). This finding is in agreement with the previous observation in pancreatic cancer where SPIN.DOC was found to be highly expressed in pancreatic cancer samples and promotes cell proliferation, invasion and migration of hepatocellular carcinoma cell [7] .

**Figure 3:**
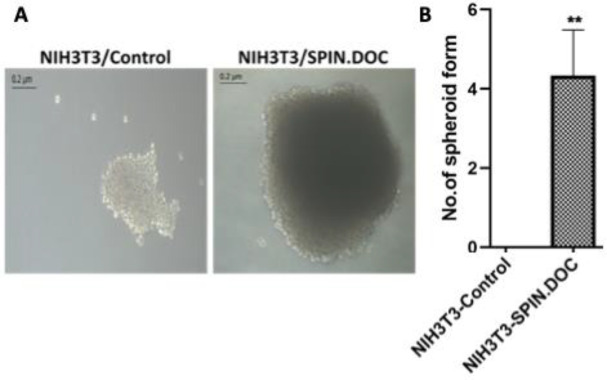
Expression of SPIN.DOC increases the spheroid ability of NIH3T3 cells.

Additionally, while NIH3T3 cells are unable to form spheroid in serum free medium, a significant number of spheroids in suspension were observed in SPIN.DOC overexpressing cells (p<0.001) (Fig. 3A and 3B). Since the ability to form floating spheroids in serum-free medium [7] is a key morphological characteristic of Cancer Stem Cells (CSCs), this suggests that SPIN.DOC overexpression may drive cellular transformation of normal NIH3T3 cells into CSCs.

**Figure 4:**
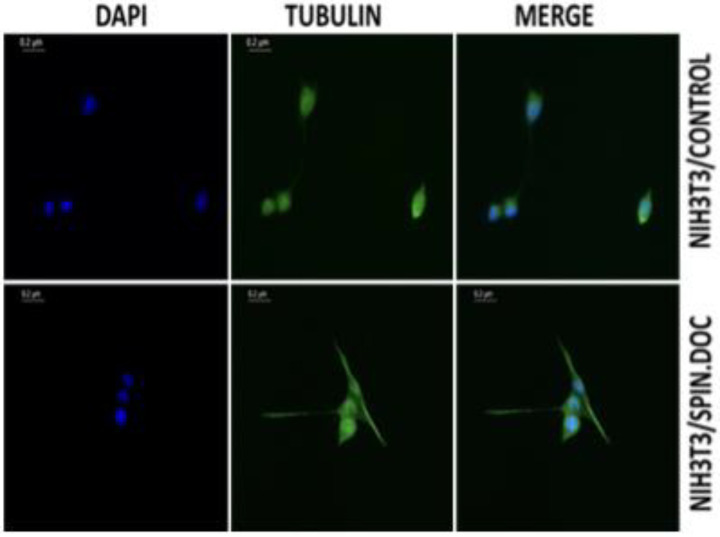
Immunofluorescence analysis of NIH3T3/Control and NIH3T3/SPIN.DOC cells with anti-β tubulin. Scale bar, 0.2 µm.

SPIN.DOC expression in NIH3T3 cells does not alter expression level of tubulin but increase the frequency of tubulin based micro tentacles (McTNs) or lamellipodia like structures protruding out, in the cytoplasm of SPIN.DOC overexpressed cells compared to the normal NIH3T3 cells (Fig. 4). This indicates that SPIN.DOC may regulate and channelized the tubulin to undergo post translational modification (PTM), potentially enhancing cell migration through epithelial mesenchymal transition (EMT), promoting proliferation and providing micro-environment niche conducive to stemness. 

(A). Representative image of NIH3T3/Control and NIH3T3/SPIN.DOC cells migrated through the Boyce chambers, stained with Giemsa stanning solution. (B) Graphical representation of the number of cells migrated. (C) Representative image of invading NIH3T3/Control and NIH3T3/ SPIN.DOC cells through the Boyce chamber (D) Graphical representation of the number of invaded cells. Data represented as the mean of ± SD of n=3 experiments,**** indicates p<0.0001.

(A). NIH3T3/control and NIH3T3/SPIN.DOC cells were seeded using the special hanging drop method as described elsewhere and incubated for 14 days. The spheroids formed were then photographed and analysed using phase contrast microscope. (B). Quantification of spheroid counts with data represented as the mean of ± SD of n=3 experiments.**significantly different from control group (p<0.01).

In conclusion, this study sought to determine whether SPIN.DOC functions as a tumour suppressor gene or an oncogene. The finding suggests that ectopic expression of SPIN.DOC in NIH3T3 fibroblast cells may drive cellular transformation, highlighting its potential oncogenic role. This transformation is likely mediated through the regulation of cytoskeleton dynamics, proliferation and cellular migration which may contribute to tumour progression. 

However, to fully establish SPIN.DOC’s oncogenic properties, further investigation is required to comprehend its role in modulating key signalling pathways associated with cellular transformation. Future study should focus on identifying specific molecular interactions and downstream targets of SPIN.DOC to determine its precise mechanism of action of tumorigenesis. 
